# Cognitive diagnosis and algorithmic cultural analysis of fourth-grade Yi students’ mathematical skills in China: A case study of several primary schools in Puge county, Liangshan prefecture

**DOI:** 10.3389/fpsyg.2023.1110950

**Published:** 2023-03-14

**Authors:** Hong Zhang, Shuang Ye, Lingke Shi

**Affiliations:** School of Mathematical Sciences, Sichuan Normal University, Chengdu, China

**Keywords:** Chinese Yi ethnic group, mathematical ability, cognitive diagnosis, DINA model, algorithmic culture

## Abstract

The Liangshan Yi Autonomous Prefecture is the largest area in China inhabited by the Yi people, and the original Yi characteristics and culture are well maintained. The Yi also have a high degree of ethnic and cultural intermingling with Tibetans, Han and other ethnic groups. The level of mathematical abilities directly determines the quality of mathematical learning of Yi students. Primary four is the stage of “concrete operations,” and is a critical point in the development of mathematical symbolic awareness. In this study, the geographical location of the school and the financial income of the township in which the school is located were used as the basis for sampling, and the DINA model was used to diagnose the mathematical ability of fourth grade students in three rural Yi primary schools in Puge County. The study found that there was individual variability in the mathematical abilities of fourth grade Yi students, with 21 different types of cognitive error patterns identified, the main ones being five. In addition, the state of knowledge of fourth grade Yi students in arithmetic revealed that their overall level of mathematical ability was low, showing a lag, with none of the knowledge attributes of arithmetic being fully mastered. Cultural differences between the Chinese and Yi languages contribute to the difficulties that Yi students have in learning mathematical operations, including differences in understanding the place value system, zero, decimal expressions, and differences in the perception of multiplication and division. The above research can inform the implementation of targeted remediation for teaching and learning.

## Introduction

### Problem statement

There are 55 ethnic minorities among China’s 56 ethnic groups. Yi is one of the ethnic minorities who still use their own language and characters, distributed in Sichuan, Yunnan, Guizhou and other places. Liangshan Yi Autonomous Prefecture is the largest Yi settlement in China, located in the south of Sichuan Province, China, with more than 1.8 million people, which is a key area in the fight against educational poverty. Mathematical operation ability is the most basic ability in mathematics, in accordance with the needs of society and the requirements of educational development, the content and training requirements for the teaching of number and calculation in curriculum standards are constantly being improved in and outside China. The fourth grade of primary school is the transition period from the lower to the upper grades. This is the period when reversible thinking is formed, a sense of conservation of concepts can be developed, and concrete logical reasoning can be made. The fourth grade is also a critical point in the development of mathematical symbolic awareness, as students begin to move from empirical to theoretical thinking (Zhu and Ma, 2018). The level of mathematical operation ability directly determines the quality of Yi students’ learning in mathematics. Studies have shown that the overall trend of Chinese students’ mathematical ability is weakening (Zhou, 2011), while in Sichuan Province, the primary school students who have difficulties in arithmetic are mainly located in rural areas and Yi ethnic groups (Zhang et al., 2009).

Since ancient times, due to the existence of natural barriers such as mountains, rivers and gullies in Liangshan area, compared with Yunnan and Guizhou, the characteristics and cultural original ecology of Yi people in this area are well maintained. At the same time, Liangshan area is within the “Tibetan-Yi-Corridor,” which is the necessary place for the ancient “Southern Silk Road.” Yi people have more cultural blending with other ethnic groups, such as Tibetan and Han. Culture has a fundamental impact on mathematics education, and the level of mathematical ability contains the elements of algorithm culture. Therefore, it is necessary to analyze the Yi algorithmic culture based on the cognitive diagnosis of mathematical ability of the fourth grade Yi students in Liangshan Prefecture, Sichuan Province, China.

### Literature review

Research on the mathematical ability of ethnic minority students is divided into three main categories. The first concerns using indicators such as correctness and frequency to portray students’ arithmetic ability. For example, Zhou et al. (2010) portrayed the school readiness of children in Guangxi Zhuang Autonomous Region (GZAR) using the number frequency index, which differed from that of children of Han ethnicity. Zuo and Tao (1994) used the question correctness index to study the current state of the arithmetic ability of 7 to 8-year-old Han and Dai students. It was found that the language barrier of Dai children made arithmetic more difficult. The second regards cross-cultural comparison by means of difference analysis. For example, Zhou et al. (2005) compared the development of arithmetic ability in 9–12-year-old Wei and Han students and found that Wei children are lagging behind by 3 years. Lu (2004) pointed out that, compared to spatial imagination and reasoning skills, the highest average score for “Wei Wei,” “Wei Han,” and “Han Han” students was in arithmetic skills. Xia (2001) compared the arithmetic ability of Buyei and Han students and showed that Han students have better arithmetic ability than Buyei, and the more Han students there are, the stronger the students’ arithmetic ability. The third concerns analysis of students’ types of arithmetic errors. For example, He et al. (2020) found that primary school students in South Xinjiang mainly showed conceptual errors in arithmetic, and the main cause was that they did not understand the algorithm and arithmetic of complex operations, the meaning of the place value system, and the like.

The first two types of research pay more attention to the accuracy and scoring rate of the questions, which are general test scores of the subjects and do not provide more detailed diagnostic information. The third kind of research analyzes the causes of the errors in the algorithm and arithmetic of ethnic minority operations. It can also further analyze the influence of ethnic minority culture on the algorithm.

### Research questions

In this study, we take fourth grade rural Yi students in Liangshan Prefecture, China as the object of study, conduct cognitive diagnosis and algorithmic cultural analysis on mathematical ability, provide reference for the implementation of targeted remedial measures in teaching, and advocate the pluralistic interaction of various ethnic cultures, leading to the conscious acceptance of cultural identity and citizenship by ethnic students (Da, 2019). Specifically, the following two research questions guided this study: (a) What is the knowledge state of arithmetic of the fourth grade Yi students in Liangshan Prefecture? (b) What are their mastery of arithmetic attributes and related cultural factors?

## Materials and methods

### Sample selection

Since the level of economic development has a significant positive correlation with educational input (Cai and Zheng, 2013), the economically underdeveloped Puge County was selected as the study area based on the monthly reports of the prefecture-wide and county and city gross domestic product of Liangshan Prefecture in the past 5 years. Puge County has a registered population of 219,000, of which 189,000 are Yi, accounting for 86.3%. It is a county inhabited by 24 ethnic minorities, mainly Yi, Han, and Hui. The 2017–2021 monthly reports of the statewide GDP of Liangshan Prefecture show that Puge County’s GDP is ranked the 14th to 16th among the seventeen counties and cities in the prefecture^1^, with a low level of economic development, insufficient investment in education, and relatively weak teaching resources.

Using a stratified sampling method, taking the geographical location of the school and the economic development of the township where the school is located as the sampling basis. All fourth grade students from three schools in three townships were selected as the sample from 34 primary schools in Puge County^2^, with a total of 7 classes and 312 fourth grade students, all of Yi ethnicity. The subjects had learned about addition, subtraction, multiplication, and division of integers and addition and subtraction of decimals before the test, which lasted 40 min. In all, 273 valid data were collected, including 82 from G school, 87 from Y school, and 104 from L school (Table 1).

**TABLE 1:** Sample selection.

School	Location	Annual revenue of the township where the school is located (million)	Effective sample size
G	Scenic area	380	82
Y	General township	136	87
L	General township	283.5	104

### Testing tools

Psychological and educational measures included the following instruments: classical test theory (CTT), item response theory (IRT), and cognitive diagnostic theory (CDT) (Mislevy and Robert, 1982). CTT only provides total test scores, merit rates, pass rates, or level classification specifics, and is concerned with student performance or where students’ current level. The main purpose of IRT is to position subjects on a trait (e.g., academic achievement, spatial ability) scale (an instrument that portrays size, how much), that is, to give an ability value, and to select and place them accordingly. They both consider the psychological trait being measured as a “statistical structure” with no clear psychological meaning, and aim to give a holistic assessment of the individual at a macro level, assigning a value that indicates a position on a unidimensional, linear, continuous system of measures (Liu et al., 2006; Tu et al., 2012). CTT and IRT measurement theories focus on the results of test scores, and although they have the advantage of providing a macro-level assessment of students’ overall ability levels, they do not provide the underlying knowledge structures behind the scores. Therefore, a cognitive diagnostic approach in a micro sense was used to test the mathematical ability of Yi fourth graders, emphasizing the combination of macro ability level and micro cognitive level assessment, providing the hidden knowledge structure status and cognitive attribute distribution behind the scores through the subjects’ responses, and explaining the reasons for the subjects’ errors (Nichols and Joldersma, 2008), thus providing targeted remediation instruction.

## Development and testing of test instruments

### Testing method

The test framework for cognitive diagnosis consists of two parts: the development of the test instrument and the test, and the use of the test instrument and the interpretation of the results (Gierl, 1997). It is necessary to first identify the cognitive attributes of mathematical operation skills and their hierarchical structure (Leighton et al., 2004) to guide the development of the test paper. Since the mathematical skills of fourth graders are an overall representation of the arithmetic knowledge learned in grades 1 to 4, the arithmetic content of primary school mathematics textbooks from grades 1 to 4 was first analyzed, which mainly included addition, subtraction, multiplication, and division of whole numbers and decimals. In-depth discussions with four current primary mathematics teachers resulted in the identification of 10 cognitive attributes of fourth-grade students’ mathematical skills (Table 2).

**TABLE 2:** Attributes of mathematical ability.

Properties	Elements
A1	In-table multiplication (1-digit by 1-digit)
A2	Multiplying multi-digit numbers by 1-digit integers
A3	Division of whole numbers with 1-digit divisors
A4	Adding whole numbers up to 10,000
A5	Subtraction of whole numbers up to 10,000
A6	Multiplying 2-digit numbers by 2-digit integers
A7	Multiplying 3-digit numbers by 2-digit integers
A8	Dividing whole numbers with 2-digit divisors
A9	Decimal addition
A10	Decimal subtraction

**FIGURE 1:**
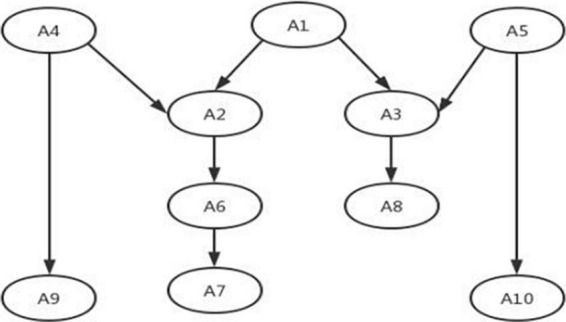
Hierarchical relationship of knowledge attributes of mathematical operations.

After determining the cognitive properties, it was necessary to classify the 10 mathematical operation properties extracted above into their hierarchical structure. The history of mathematical development shows that different categories of operations are formed and developed gradually from simple to complex, concrete to abstract, and low to high level. Therefore, the knowledge and mastery of operations must also be progressively ordered and hierarchical (Wynn, 1992; Von Aster, 2000). Students were asked to elaborate their arithmetic processes in the form of oral reports, and the opinions of current mathematics teachers were sought to delineate the cognitive order of the above 10 arithmetic attributes, and then to verify the rationality of the attribute hierarchy using the hierarchy consistency index (HCI) values in cognitive diagnostic (Cui et al., 2007; Cui and Leighton, 2009). The final hierarchical relationship diagram of the attributes of mathematical operations knowledge for grades 1 to 4 was determined (Figure 1).

### Development of the test instrument

First, the preparation of the test instrument requires the determination of the reachable matrix R describing the hierarchical structure of attributes of mathematical operation ability, which reflects all relations between cognitive attributes, including direct, indirect, and self-relations. It is a prerequisite for achieving an accurate diagnosis of each attribute. According to the reachable matrix R, with the help of the expansion algorithm, it is possible to obtain the ideal measurement model (Ding et al., 2012), that is, all test question types that satisfy the relationships of the attribute hierarchy.

The reachable matrix R cannot be obtained directly through the attribute hierarchy relationship graph, but it can be obtained with the help of graph theory knowledge. First, the adjacency matrix A corresponding to the mathematical operation knowledge attribute hierarchy relationship graph is given, based on the Boolean algorithmR = (A + I)^*n*^, where I is the unit matrix, and then flexCDMS software is used to obtain the reachable matrix R. With the increasing number of n inR = (A + I)^*n*^, when the value of R is stable and constant, then R is a reachable matrix (Table 3).

**TABLE 3:** R matrix under attribute hierarchy relations.

	A1	A2	A3	A4	A5	A6	A7	A8	A9	A10
A1	1	1	1	0	0	1	1	1	0	0
A2	0	1	0	0	0	1	1	0	0	0
A3	0	0	1	0	0	0	0	1	0	0
A4	0	1	0	1	0	1	1	0	1	0
A5	0	0	1	0	1	0	0	1	0	1
A6	0	0	0	0	0	1	1	0	0	0
A7	0	0	0	0	0	0	1	0	0	0
A8	0	0	0	0	0	0	0	1	0	0
A9	0	0	0	0	0	0	0	0	1	0
A10	0	0	0	0	0	0	0	0	0	1

Once the reachable matrix R is obtained, 71 test question types that satisfy the structural relationship of the attribute hierarchy can be obtained using the expansion algorithm. When the test question types contain the reachable matrix R, it is possible to accurately diagnose each knowledge attribute to be examined, making the examination of the attributes structurally valid (Ding et al., 2011). Of the 71 question types, only 10 overlapped completely with the R matrix. The initial test prepared 43 questions based on the 10 question types in the R matrix, and after eliminating 7 questions with low discrimination, 36 questions were finally formulated. Typically, test questions should be developed to enable multiple measures of each attribute, so that each attribute is measured at least three times (Tu et al., 2012).

Second, the Q-matrix describing the relationship between the questions and cognitive attributes of the whole paper needs to be obtained (Tatsuoka et al., 1995). The Q matrix corresponding to the 36 questions shows whether the attribute was examined for each question, where 1 is examined and 0 is not examined (Lawrence and DeCarlo, 2012; Feng et al., 2014; Table 4). Thus, the Q matrix was obtained for all test questions that satisfy the R matrix. The test questions were selected from the 2014 to 2020 Liangshan Prefecture 4th grade mathematics final exam question bank and prepared in consultation with teaching researchers and current teachers. Two types of questions are included: oral computation and columnar computation. There are 21 oral calculations and 15 vertical calculations. All questions are scored from 0 to 1, with 1 point per question, for a total of 36 points.

**TABLE 4:** Q matrix corresponding to 36 questions.

	T1	T2	T3	T4	T5	T6	T7	T8	T9	T10	T11	T12	T13	T14	T15	T16	T17	T18	T19	T20	T21	T22	T23	T24	T25	T26	T27	T28	T29	T30	T31	T32	T33	T34	T35	T36
A1	1	1	1	1	1	1	0	0	1	1	0	1	0	0	0	1	1	1	1	0	1	0	0	0	0	0	0	1	1	1	1	1	1	1	1	0
A2	0	1	1	1	0	0	0	0	0	0	0	1	0	0	0	0	0	1	1	0	1	0	0	0	0	0	0	1	1	1	1	1	0	0	0	0
A3	0	0	0	0	1	1	0	0	1	1	0	0	0	0	0	1	1	0	0	0	0	0	0	0	0	0	0	0	0	0	0	0	1	1	1	0
A4	0	1	1	1	0	0	1	1	0	0	1	1	0	0	0	0	0	1	1	1	1	0	1	1	0	0	1	1	1	1	1	1	0	0	0	0
A5	0	0	0	0	1	1	0	0	1	1	0	0	1	1	1	1	1	0	0	0	0	1	0	0	1	1	0	0	0	0	0	0	1	1	1	1
A6	0	0	0	0	0	0	0	0	0	0	0	1	0	0	0	0	0	1	1	0	0	0	0	0	0	0	0	1	1	1	1	1	0	0	0	0
A7	0	0	0	0	0	0	0	0	0	0	0	1	0	0	0	0	0	1	0	0	0	0	0	0	0	0	0	1	0	1	0	1	0	0	0	0
A8	0	0	0	0	0	0	0	0	0	0	0	0	0	0	0	1	1	0	0	0	0	0	0	0	0	0	0	0	0	0	0	0	1	1	1	0
A9	0	0	0	0	0	0	0	0	0	0	0	0	0	0	0	0	0	0	0	1	0	0	0	1	0	0	1	0	0	0	0	0	0	0	0	0
A10	0	0	0	0	0	0	0	0	0	0	0	0	0	0	1	0	0	0	0	0	0	0	0	0	1	1	0	0	0	0	0	0	0	0	0	1

### Testing of test instruments

Once the test paper is prepared, its quality needs to be checked against the measurement requirements of reliability, validity, completeness of attributes, and discrimination. According to flexCDMs calculations, the cognitive diagnostic reliability of each arithmetic attribute in the test paper is >0.75, showing good individual reliability. In addition, the average reliability is 0.9053, which indicates good overall reliability. For the validity of the test papers, since the development of cognitive diagnostic tests is done on the premise of attributes and attribute hierarchical relationships, the scientific soundness of the test directly depends on the soundness of the hierarchical relationships between attributes. Gierl et al. (2008) suggested that as long as the HCI is higher than 0.70, it indicates that a good cognitive hierarchical model has been constructed; that is, the assumed hierarchical structure of attributes is sound. The HCI of the test paper was calculated according to flexCDMs = 0.7426 > 0.70, and thus the structural validity was good. In examining the completeness of the 10 selected attributes, a regression analysis was created with question difficulty as the dependent variable and arithmetic attributes as the independent variables, and all cognitive attributes were calculated to explain 82.2% of question difficulty with an effect value of 0.761, all greater than 60%. The knowledge attributes included in the test explained more than 60% of the difficulty, which means that the knowledge attributes selected for the test are complete (Junker and Sijtsma, 2001); thus, the 10 cognitive attributes finally identified in the test paper are more scientific and reasonable, and can reflect the complete knowledge of mathematical operations learned by the fourth-grade students. The results of the analysis of the R-linguistic platform show that the average differentiation value of the 36 test questions is 0.57. In L. Ebel’s opinion, according to the criteria that can be proposed to evaluate the merits of the questions according to the discrimination index (Ding, 2001), a discrimination of 0.4 or more indicates a very good question, a discrimination of 0.3 to 0.39 indicates a good question, and a discrimination of 0.2 to 0.29 indicates a fair question. Only one of the 36 test questions has a discrimination below 0.2, indicating that the discrimination of the test items is generally good.

## Results

Existing cognitive diagnostic models can be divided into two categories: latent trait models (Fischer, 1973) and potential classification models (Tatsuoka, 1983). The latent trait model aims to analyze what latent traits the subject has, based on the scores obtained from the subject. Latent classification models are designed to classify the subject and help the subject find its place in the group. This test uses the DINA model (Henson, 2005; Wu et al., 2020), which is a latent classification model that allows the data collected from student responses to be processed to analyze the individual’s knowledge status and mastery of cognitive attributes behind the scores (de la Torre, 2011). Compared to other potential classification models, the DINA model not only portrays the mental processes of students during arithmetic, but also involves only two parameters, the miss parameter and the guess parameter, which are easy to implement for estimation of these two parameters. Therefore, it is a concise and easy to explain model with the following advantages: both its miss and guess parameters are calculated based on the question level, and the complexity of the model is not affected by the number of attributes. Despite its simplicity, this model has been shown to have a good model fit (de la Torre and Douglas, 2004) and is an excellent model in cognitive diagnosis, which is widely used in practice.

### Analysis of state of knowledge

The DINA cognitive diagnostic model for Yi fourth graders’ mathematical ability allows us to obtain each student’s arithmetic knowledge status (Tatsuoka, 2009). The DINA diagnostic model was used to diagnose each student’s arithmetic knowledge, with a “1” indicating that the student mastered the attribute and a “0” indicating that the student did not master the attribute. The diagnosis of the knowledge status of mathematical ability of students in all three schools reflects the existence of individual differences among students (Table 5).

**TABLE 5:** A total of 4th-grade Yi students’ mathematical skills and cognitive diagnostic knowledge state.

School	Class	Name	Sex	State of knowledge (attribute mastery model)									
				A1	A2	A3	A4	A5	A6	A7	A8	A9	A10
Y	4.1	Ji***Xia	Female	1	1	1	1	1	1	1	1	1	1
Y	4.1	Jie**Xia	Male	1	1	1	1	1	1	1	0	1	0
Y	4.2	A***Zuo	Female	1	1	1	1	1	0	1	0	1	1
Y	4.2	E**Xia	Male	1	1	0	1	1	0	1	0	1	0
…													
G	4.1	Ji***Wai	Female	1	1	0	1	1	1	1	0	1	0
G	4.1	Ri** Dao	Male	1	1	1	1	1	1	1	0	1	0
G	4.2	Nai**Xia	Male	1	0	0	1	1	0	0	0	1	0
G	4.2	La***Zha	Female	1	1	0	1	0	1	1	0	1	0
…													
L	4.1	A**Qu	Male	1	1	0	1	1	1	1	0	1	1
L	4.1	La**Cong	Male	1	1	0	1	1	0	1	0	1	0
L	4.2	Bao***Niui	Female	1	1	0	1	0	0	0	0	0	0
L	4.2	Ji***Za	Female	1	1	0	1	1	1	1	0	1	0
L	4.3	A*** Li	Female	1	1	0	1	1	1	1	0	1	1
L	4.3	Shi*** Niu	Female	1	1	1	1	1	1	1	1	1	1
…													

For example, the arithmetic knowledge state of Xie**Xia is “11111111010,” while the arithmetic knowledge state of Nai**Xia is “1001100010.” Based on the cognitive diagnostic knowledge state table, teachers can grasp each student’s strengths and weaknesses in arithmetic and teach them according to their needs. For example, A**Qu’s knowledge state is “1101111011,” which means that the student has not yet mastered the division of integers by one digit (A3) and the division of integers by two digits (A8); therefore, the teacher needs to strengthen the remedial learning of attributes A3 and A8 for targeted homework. ***Indicates the first name in the student’s name.

Based on the diagnostic results of the DINA model, the subjects’ knowledge states were obtained by the great *a posteriori* estimation MAP calculation method, and then matched with the 72 ideal mastery patterns already obtained. It was found that 22 of the subjects’ knowledge mastery states were in the ideal mastery pattern (Table 6).

**TABLE 6:** Classification of subjects’ ideal mastery patterns.

State of knowledge	Frequency	Percentages	State of knowledge	Frequency	Percentages
1101000000	7	2.56%	1001000010	3	1.10%
0001000000	7	2.56%	0001100000	1	0.37%
1101011000	3	1.10%	1001100000	4	1.47%
1101110000	2	0.73%	1101111000	11	4.03%
0001100010	1	0.37%	1001100010	8	2.93%
1101010010	1	0.37%	1111111100	3	1.10%
1101011010	8	2.93%	1111111010	7	2.56%
1111111001	1	0.37%	1101111010	6	2.20%
1111110101	3	1.10%	1111111101	3	1.10%
1111110101	2	0.73%	1101111011	14	5.13%
1111111011	25	9.16%	1111111111	120	43.96%

According to the above classification table, 240 out of 273 subjects whose mastery patterns conformed to the corresponding hierarchical structure relationship in Figure 1 were successfully classified as having ideal mastery patterns, with a high rate of 87.91%. As can be seen from Table 5, except for the full mastery pattern (11111111111), all the other 21 patterns indicated that Yi fourth graders made different types of cognitive errors, mainly “111111011,” “1101111011,” “1101111000,” “1001100010,” and “1101011010,” which are the five types, constituting 24.18% of the total number and 43.15% of the total cognitive errors.

In addition, the fourth grade Yi students showed a lag in their arithmetic skills. The subjects with the knowledge status of “0001000000,” “0001100000,” and “1001100000” were at the 2nd grade level, while those with the knowledge status of “1101000000” and “1101110000” were at the 3rd grade level. The subjects with the knowledge status of “1101000000” and “1101110000” were at the level of grade 2, and those with the knowledge status of “1101000000” and “1101110000” were at the level of grade 3. A total of 60.08% of the fourth grade Yi students mastered all the arithmetic knowledge (A1∼A6) learned in grades 1–3, and only 43.96% mastered all the arithmetic knowledge (A1–A10) learned in grades 1–4.

### Analysis of cognitive attribute mastery

According to the mastery statistics of each arithmetic attribute (Table 7), the overall level of mathematical ability of the fourth-grade students in Liangshan Yi Prefecture is low, and none of the ten arithmetic knowledge attributes has been fully mastered, among which integer addition within 10,000 (A4) is the best mastered, and integer division with 2-digit divisors (A8) is the worst mastered.

**TABLE 7:** Cognitive attribute mastery statistics.

Property code	Number of attribute holders	Percentage of attribute mastery
A1	259	94.87%
A2	249	91.21%
A3	185	67.77%
A4	264	96.70%
A5	239	87.55%
A6	209	76.56%
A7	225	82.42%
A8	132	48.35%
A9	214	78.39%
A10	181	66.30%

**FIGURE 2:**
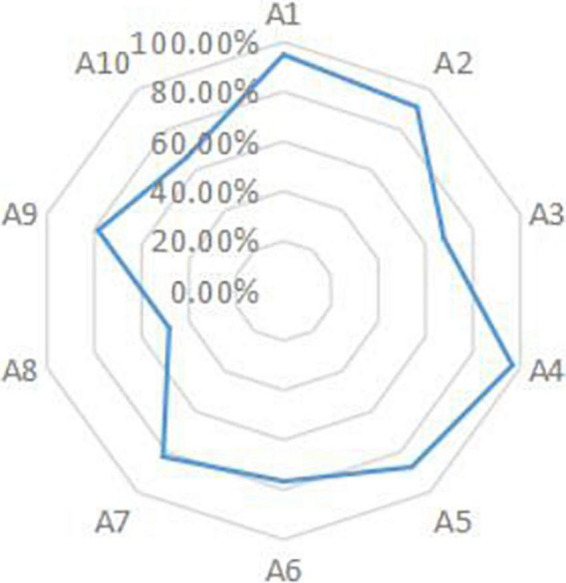
Radar chart of student knowledge attribute mastery.

Among the operations A1 to A6 studied in grades 1 to 3, in-table multiplication (A1), multiplication of multi-digit numbers by 1-digit numbers (A2), addition of integers within 10,000 (A4), and subtraction of integers within 10,000 (A5) were relatively well mastered, but these six attributes were not fully mastered by students, indicating that Yi fourth graders had “underachieved” when it comes to the arithmetic knowledge learned in grades 1 to 3. Figure 2 is a visual representation of the radar chart.

The less than satisfactory mastery of the property of arithmetic (A7 to A10) by Yi fourth graders could be attributed to the failure to master the arithmetic knowledge learned in grades 1 to 3 (A1 to A6). Division of integers with 2-digit divisors (A8) studied in grade 4 had the lowest mastery rate of 48.35%, that is, more than half of the students did not master this property. The reason for this is that its prerequisites are mastery of the knowledge attribute in-table multiplication (A1), division by whole numbers with 1-digit divisors (A3), and subtraction of whole numbers up to 10,000 (A5), and as many as 49.81% did not master the prerequisites, resulting in subjects who did not master A1, A3, and A5 also failing to master division by whole numbers with 2-digit divisors (A8). Similarly, students at this grade level had difficulty in decimal operations, with mastery of decimal addition (A9) (78.39%) and decimal subtraction (A10) (66.30%) ranking in the bottom five and bottom two, respectively.

### Summary of main results

Cognitive diagnosis through the DINA model leads to the following main outcomes.

First, the state of knowledge of fourth grade Yi students in arithmetic is captured. Students’ mastery of ten cognitive attributes is accurately described, and individual differences in the mathematical abilities of students scoring the same are then identified as a means of targeting training and remediation.

Second, all cognitive error patterns and major cognitive error patterns in arithmetic were obtained for fourth grade Yi students. The knowledge states obtained from the diagnosis were matched with 72 ideal mastery patterns to obtain 21 different types of cognitive error patterns, which in turn led to the identification of the main five error patterns, accounting for 43.15% of the total cognitive errors.

Third, the overall mastery of the properties of arithmetic knowledge by fourth grade Yi students was understood. The overall level of mathematical ability of fourth grade Yi students in Liangshan Prefecture was low, and none of the knowledge attributes of arithmetic were fully mastered. A total of 60.08% of the fourth grade Yi students mastered all of the arithmetic knowledge learned in grades 1 to 3, while only 43.96% mastered all of the arithmetic knowledge learned in grades 1 to 4, showing an overall lag in arithmetic skills.

## Discussion

### Differences in traditional Yi cultural understanding of the place value system, zero, and decimal expressions

**FIGURE 3:**
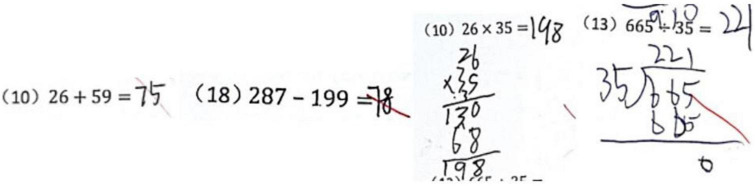
Reminding error, debit error, spatial arrangement error, failure to consider divisor as a whole.

From a semiotic point of view, all real mathematical activities are related to the representation of mathematical objects rather than to the mathematical objects themselves (Otte, 2006). The students’ relationship to reality is mediated by symbols or symbolic processes. The errors made by the fourth grade Yi students in performing the four operations were related to the representation and understanding of the place value system. The following were the errors in operations: rounding errors in addition, debit errors in subtraction, spatial arrangement errors and rounding errors in multiplication, and failure to consider the divisor as a whole and misplacement of the quotient in division (Figure 3). All reflect barriers to Yi fourth graders’ representation and understanding of the place value.

The Yi language differs from Han Chinese notation in that the Yi language does not have a place value system. The “place value system” refers to the fact that the same symbol is assigned a different value depending on its relative position in the numerical representation. The Yi word for “eleven” is written as “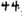,” not a direct spelling of “ten” and “one,” which implies the idea of “ten plus a starting number.” Similarly, when “twenty” is expressed in Yi, it is not “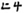,” but “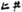,” which means “two plus another ten.” In addition, the number “11” is written as “
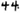
” when expressed in Yi, but the number “1” is expressed as “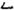.” The number “20” is written as “
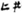
” in Yi, but the number “2” is represented as “
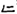
” (Wang, 2018). The above notation indicates that the Yi algorithm does not have a place value system, which leads to barriers to understanding the concept of place value system for Yi students.

**FIGURE 4:**
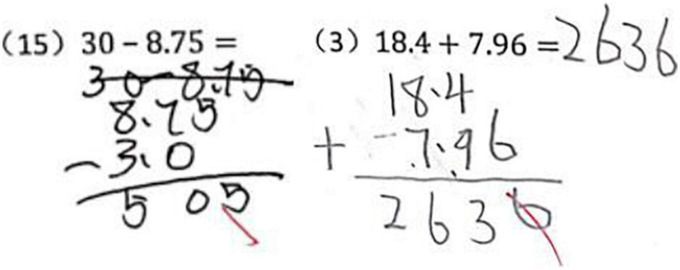
Improper understanding of decimal concepts and omission of decimal point.

The absence of “zero” and “decimal expressions” in Yi algorithms is also a major obstacle for Yi students to learn decimal operations. Usually, even when people of one ethnicity learn the language of another people, they must translate themselves in their own heads when they learn the culture of that ethnicity (Yi and Ye, 1996). Fourth graders of the Yi ethnic group also translate from Chinese to Yi when they learn mathematical operations and think in Yi. However, the Yi do not have a word for “zero” or an expression for decimals. Interviews with Yi students revealed that they use different units to represent numbers in different digits; for example, 10.09, which is pronounced as “ten point zero nine” in Chinese, is expressed in Yi as “10 yuan and 9 cents,” omitting the “zero” and “decimal point.” According to the results of the cognitive diagnostic assessment, the probability of mastering decimal addition and subtraction for Yi fourth graders was only 78.39 and 66.30%, respectively, and the students’ misunderstanding of decimal concepts and omission of decimal points (Figure 4) were related to the lack of “zero” and “decimal expression” in Yi algorithms.

The place value system, zero sign, and decimal expressions in the number system all involve symbolic meaning, and constructing meaning for these symbols requires a complex process of negotiation through discourse (Presmeg, 2006). In teaching arithmetic to Yi students, teachers can create an algorithmic symbol system based on ethnic culture and use a semiotic chain model of instruction to create a series of abstract concepts, while preserving important relationships in students’ everyday practice, to help Yi students build a complete cognition of the number system.

### Differences in Yi-Han algorithmic culture lead to different perceptions of multiplication and division

**FIGURE 5:**
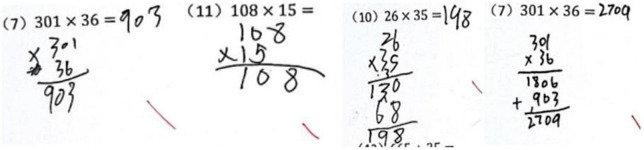
High alignment and start-from-high-bit arithmetic error, recede-to-the-right error.

Yi fourth graders all had problems with spatial misalignment when multiplying integers: that is, three-digit by two-digit integer multiplication where the multiplier is aligned with the first digit of the multiplied number and only the higher digit of the multiplier is counted in vertical calculations, or two-digit by two-digit integer multiplication (three-digit by two-digit integer multiplication) where the multiplication tens digit calculation is shifted one place to the right (Figure 5). In Han multiplication, the multiplier is aligned with the last digit of the multiplied number, and each digit of the multiplied number is multiplied by the last digit of the multiplied number. The product of the second and subsequent multiplications is shifted one digit to the left. However, in Yi multiplication, the multiplier is aligned with the highest digit of the multiplied number, and the digit in the highest digit of the multiplied number is multiplied by each digit of the multiplied number. The product of the second and subsequent multiplications is shifted one digit to the right. The second and subsequent products are shifted one place to the right, which is different but the result is the same (Wu et al., 1996).

Mathematical activities require different symbolic representation systems. This highlights a key issue in mathematical understanding: how learners can identify the same representational objects through symbolic representations produced by different representational systems (Duval, 2006). Yi fourth graders either used the Yi algorithm but incomplete calculations when performing multi-digit multiplication operations or confused the use of the Yi multi-digit multiplication algorithm with the Chinese multi-digit multiplication algorithm. Since the two operations are exactly opposite, this confused the thinking of Yi fourth graders in multi-digit multiplication operations and resulted in different arithmetic errors. Therefore, Yi teachers should focus on explaining to students the differences and connections between the Yi and Chinese algorithmic cultures when teaching multi-digit multiplication operations.

**FIGURE 6:**
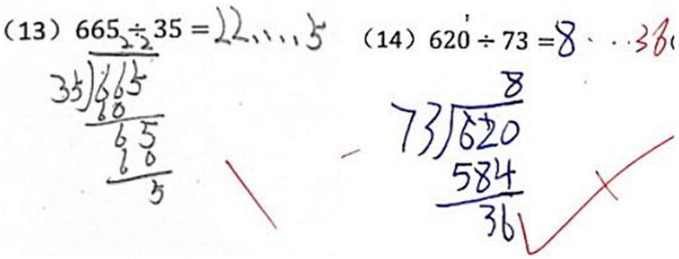
Confusing division operations for addition, subtraction and multiplication operations, not estimating quotients correctly, missing remainder errors.

Yi fourth graders had the poorest mastery of division, especially division by whole numbers with two-digit divisors. Typical errors of Yi fourth graders in division operations included confusing division operations with addition, subtraction, and multiplication operations; not estimating quotients correctly; and missing remainders (Figure 6). Yi minorities commonly try to multiply and subtract to roughly solve some simple division, and often ignore the remainder when they cannot divide whole (Wei and Qin, 2014). Fourth-grade Yi students are more unfamiliar with division operations than with other operations; therefore, teachers should focus on teaching students the differences between division operations and addition, subtraction, and multiplication operations, and on developing students’ estimation skills to lay the foundation for them to be able to estimate quotients correctly. For division with remainders, teachers should explain the origin of the remainder to students to avoid the error of ignoring the remainder.

The history of the development of mathematics shows that the development of symbolic representations is necessary for the development of mathematical thought. Mathematical objects beginning with numbers are not objects that can be directly perceived or observed, and the learning of numbers must be based on a system of representations. The problems and difficulties in minority mathematics pedagogy can be understood to some extent in terms of cultural conflicts (Bishop, 1994). Therefore, it is necessary to study both the acquisition and transmission of culture in the teaching of mathematics, to continuously improve the process of instructional design and implementation, and to effectively improve the quality of teachers’ knowledge construction systems.

## Conclusion

Native languages have a significant impact on people’s ability to process mathematical problems, and languages representing different cultural backgrounds can affect the way people’s brains process mathematical information (Tang et al., 2006). There are many differences between Yi and Han in terms of sentence structure. For example, Han Chinese uses subject-verb-object expressions, while Yi uses subject-verb-predicate expressions. Interviews with fourth-grade Yi students revealed that Yi students would read questions in a different order than in Han Chinese, which resulted in their inability to understand the meaning of the question. For example, “3 minus 2” is expressed in Yi as “32 minus,” “6 plus 7” is expressed in Yi as “67 plus,” and “4 times 5” is expressed in Yi as “45 times.” Thus, the cultural differences between Chinese and Yi lead to difficulties for students in learning mathematical operations.

As Yi language mathematics teachers suffer from a lack of ontological knowledge base of mathematics subjects, insufficient multicultural integration skills, weak professional development skills, and ambiguous professional development beliefs, students mostly rely on their personal linguistic translations to understand abstract mathematical knowledge. The generally low Chinese language level of Yi students makes it inevitable that some incorrect translations will occur during the conversion from Chinese-mathematical language-Yi language, resulting in a misunderstanding of mathematical knowledge, theorems, formulas, logic, etc., which reduces the effectiveness of learning (Li, 2012). From the aspects of mathematical subject content compensation, mathematical problem identification in multiple contexts, and ethnicized material mining for teaching design, it is essential to facilitate the professional knowledge development of minority mathematics teachers and to improve the arithmetic ability of minority students (Sun and Zhang, 2020).

## Data availability statement

The raw data supporting the conclusions of this article will be made available by the authors, without undue reservation.

## Ethics statement

The studies involving human participants were reviewed and approved by the Sichuan Normal University. Written informed consent to participate in this study was provided by the participants’ legal guardian/next of kin.

## Author contributions

HZ and SY: conceptualization, methodology, formal analysis, writing—original draft preparation, and several rounds of revision. SY and LS: data collection and editing. HZ and LS: writing—subsequent drafts, reviewing, revising, proofreading, and final draft, and preparation and editing for submission. HZ: supervision and project administration. All authors contributed to the article and approved the submitted version.

## Footnotes

1 Statistics based on data published by the Liangshan Yi Autonomous Prefecture Bureau of Statistics for 2017–2021.

2 According to the ‘Directory of Schools in Puge County’ published by the People’s Government of Liangshan Yi Autonomous Prefecture in 2022.
